# GABA and glutamate measurements in temporal cortex of autistic children

**DOI:** 10.1002/aur.3253

**Published:** 2024-11-11

**Authors:** Muhammad G. Saleh, Luke Bloy, Lisa Blaskey, Timothy P. L. Roberts

**Affiliations:** ^1^ Lurie Family Foundations MEG Imaging Center, Department of Radiology Children's Hospital of Philadelphia Philadelphia Pennsylvania USA; ^2^ Department of Radiology, Perelman School of Medicine University of Pennsylvania Philadelphia Pennsylvania USA

**Keywords:** autism, GABA, glutamate, MEGA‐PRESS, MM‐suppressed

## Abstract

Autism spectrum disorder (ASD) is a neurodevelopmental disorder and presents with challenges in social communication. A hypothesized underlying contributing mechanism is the imbalance in excitation and inhibition (E/I), partly influenced by the levels of excitatory neurotransmitter glutamate (Glu) and inhibitory neurotransmitter γ‐aminobutyric acid (GABA) in the brain. Although many have reported the levels of GABA and Glu in the brain, only a few reports address the temporal cortex and then only with a small sample of autistic children, and often only in one hemisphere. We used a macromolecular suppressed edited‐magnetic resonance spectroscopy (MRS) sequence to study GABA and Glu (as potential key players influencing E/I) in a large sample of children with ASD in the right and left temporal cortices of children with (*N* = 56) and without (*N* = 30) ASD (7–18 years). As a group, children with ASD exhibited no differences in the left hemisphere (GABA and Glu Cohen's |d|: 0.24 and 0.03), but the right hemisphere showed higher GABA and lower Glu concentrations (GABA and Glu Cohen's |d|: 0.53 and 0.65) compared to neurotypicals. Furthermore, a negative association was found between the right hemisphere Glu levels of the ASD group and a clinical assessment tool (r = −0.361, *p* = 0.022), reflecting autism trait severity (social responsiveness scale). In conclusion, we highlight the chemical abnormalities in children with ASD through a cross‐sectional measurement. Longitudinal studies are warranted to determine whether these chemical levels persist or resolve over development.

## Lay Summary

Autism spectrum disorder (ASD) is a brain developmental disorder possibly affected by an imbalance of the brain chemicals γ‐aminobutyric acid (GABA) and glutamate (Glu). In this study, we assessed the levels of these chemicals in the left and right sides of the brain that are responsible for language abilities. We found changes in the GABA and Glu levels of the ASD population, and Glu in ASD was associated with the social impairment associated with ASD.

## INTRODUCTION

Autism spectrum disorder (ASD) is a neurodevelopmental disorder presenting challenges with social communication and restricted or repetitive behaviors (Screening and Diagnosis of Autism Spectrum Disorder, 2022). The current estimate of prevalence of ASD is ~1 in 100 worldwide and 1 in 36 in the United States (Maenner et al., 2021; Zeidan et al., 2022). Despite recognized phenotypic heterogeneity, a common biological mechanism hypothesized to underlie at least a subset of the ASD population is an imbalance in excitation and inhibition (E/I), supported by postmortem studies (Casanova et al., 2006; Fatemi et al., 2009) and in vivo magnetic resonance spectroscopy (MRS) studies (Gaetz et al., 2014; Harada et al., 2011; Port et al., 2017).

E/I balance can rely on many metabolites, involving an intricate play of multiple brain chemicals that have different suggestive roles, including N‐acetylaspartate (NAA) reporting on neuronal health, choline‐containing compounds (Cho) on neuroinflammation, and creatine‐containing compounds (Cr) on energy buffer during increased energy demands (Aoki et al., 2012). In addition to these brain chemicals, the postulated E/I mechanism in ASD can be characterized by the excitatory neurotransmitter glutamate (Glu) and inhibitory neurotransmitter γ‐aminobutyric acid (GABA). Many ASD studies have measured GABA and Glu levels in different brain regions, including the frontal lobe, lenticular nuclei (LN), motor, visual, and auditory/temporal cortices as potential key players influencing neurotransmission and E/I balance (Gaetz et al., 2014; Harada et al., 2011; Puts et al., 2017). Specifically, the temporal cortex encompasses the superior temporal gyrus (STG), which houses the auditory processing and conduit to the receptive language areas of the posterior STG (pSTG), providing speech perception and production (Buchsbaum et al., 2001).

MR imaging (MRI) and spectroscopy (MRS) have been applied to study the temporal cortex of the ASD population. Using MRI, the temporal cortex (specifically STG) of the autistic population (young children) has been reported to be hypo‐perfused (Zilbovicius et al., 2000) and may display atypical functional connectivity with other brain regions for processing language (Xiao et al., 2023). This atypical connectivity between STG and other regions supporting language processing did not show even trends toward association with better language abilities in the ASD group compared with the strong association found in the typically developing (TD) group (Xiao et al., 2023). Another study measuring cortical volume found that children with autism had a thinner STG compared with TD children, suggesting abnormal cortical growth (Wallace et al., 2010). These studies indicate that the temporal cortex (especially STG) is implicated in autism, affecting language processing. MRS studies have measured GABA and Glu in the temporal cortex (engulfing the STG area) of the ASD population to assess their levels and potential impact on E/I balance. Table 1 tabulates these results measured in the temporal cortices of adults and children with ASD, demonstrating either a decrease, increase, or no change. Rojas et al., (Rojas et al., 2014), Gaetz et al. (Gaetz et al., 2014), and Port et al. (Port et al., 2017) found lower GABA in the LH of the ASD group, whereas Kolodny et al. (Kolodny et al., 2020) and Edmondson et al. (Edmondson et al., 2020) found no GABA change in the LH and right hemisphere (RH), respectively. Devito et al. (DeVito et al., 2007) found lower Glu in the LH, and Joshi et al. (Joshi et al., 2013) and Edmondson et al. (Edmondson et al., 2020) had the same finding in the RH. Brown et al. (Brown et al., 2013) found higher Glu in both hemispheres, but Kolodny et al. (Kolodny et al., 2020) found no change in the LH. GABA and Glu alterations in some studies may have arisen from a period of atypical cortical growth (Wallace et al., 2010), impacting E/I balance that may account for communication deficits in autism (Pizzarelli & Cherubini, 2011). However, these results differ in direction (or lack thereof) probably due to the small sample enrolled in these studies, group differences reported using total creatine normalization compared with the water‐scaled concentrations, hemispheric differences, population heterogeneity, dissimilar age groups, and differing MRS methods employed in measuring these chemicals (edited vs. unedited MRS).

**TABLE 1:** GABA and glutamate (or Glx, glutamate + glutamine) alterations in the temporal cortex of individuals with ASD.

Reference	Number of subjects per group (mean ± SD age or range in years)	Children/adults/both	Methods	Glu or Glx	GABA
DeVito et al., 2007	26 ASD (6–17) vs. 29 TD (6–16)	Children	MRSI	Trend ASD < TD, mostly in LH (arbitrary units)	
Brown et al., 2013	13 ASD (36.89 ± 6.80) vs. 15 TD (41.08 ± 6.77)	Adults	PRESS	Signif. ASD > TD LH and RH (institutional units)	
Joshi et al., 2013	7 ASD vs. 7 TD (overall 12–17)	Children	2D JPRESS	Trend ASD < TD RH (institutional units)	
Rojas et al., 2014	17 ASD (14.01 ± 5.18) vs. 17 TD (12.44 ± 5.20)	Children	MEGA‐PRESS		Signif. ASD < TD LH (relative to tCr)
Gaetz et al., 2014	17 ASD (11.50 ± 2.70) vs. 17 TD (13.30 ± 2.87)	Children	MEGA‐PRESS		Signif. ASD < TD LH (relative to tCr)
Port et al., 2017	42 ASD vs. 32 TD (overall 6 to 40)	Both	MEGA‐PRESS		Trend in Adults and Signif. in children ASD < TD LH (relative to tCr)
Kolodny et al., 2020	31 ASD (22.70 ± 3.60) vs. 40 TD (23.0 ± 3.50)	Adults	MEGA‐PRESS	NS LH (institutional units and relative to tCr)	NS LH (institutional units and relative to tCr)
Edmondson et al., 2020	21 ASD (11.8 ± 1.2) vs. 20 TD (11.3 ± 1.7)	Children	sLASER for Glx MEGA‐sLASER for GABA	Signif. ASD < TD RH (institutional units)	NS RH (institutional units)
Present study	56 ASD (12.15 ± 2.65) vs. 30 TD (12.06 ± 2.94)	Children	MM‐suppressed MEGA‐PRESS	NS LH Signif. ASD < TD RH (institutional units)	NS LH Signif. ASD > TD RH (institutional units)

Abbreviations: ASD, Autism spectrum disorder; LH, left hemisphere; NA, not available; RH, right hemisphere; Signif, statistically significant (*p* ≤ 0.05); tCr, total creatine; TD, typically developing; Trend, 0.05 < *p* ≤ 0.1.

GABA can be estimated using the standard, unedited point‐resolved spectroscopy (PRESS) sequence (Bottomley, 1987). However, at clinical field strengths (≤3 T), low‐concentration GABA is largely obscured by high‐concentration Cr due to limited chemical shift dispersion in the traditional MRS spectrum (or unedited spectrum). To address the challenge, some MRS studies (Edmondson et al., 2020; Gaetz et al., 2014; Port et al., 2017; Rojas et al., 2014) adopted the MEGA‐edited scheme (Mescher‐Garwood point‐resolved spectroscopy, MEGA‐PRESS (Mescher et al., 1998), or MEGA‐semi‐adiabatic localization by adiabatic selective refocusing, MEGA‐semiLASER (Andreychenko et al., 2012)), to exploit the scalar coupling properties of GABA by selectively editing the signal in the MRS spectrum and removing the overlapping Cr signal. With the Glu signal embedded in the MEGA‐edited data, this method allows the characterization of both chemicals—GABA and Glu—in the brain.

MEGA‐PRESS application can differ between studies, depending on the positioning of the frequency‐selective (MEGA or editing) pulses. These pulses are either applied at 1.9 and 7.5 ppm to result in GABA contaminated with underlying macromolecular (MM) signal (i.e., commonly referred to as GABA+) or 1.9 and 1.5 ppm to reduce the MM contamination (Henry et al., 2001). Differences in Glu among the reported studies can also stem from the differing methods used for measuring it – either modeling the Glx signal (with Glu, glutamine, and glutathione overlapping) from the resulting GABA‐edited (or difference) spectrum, using the OFF spectrum of the MEGA data (equivalent to the standard PRESS spectrum acquired at the MEGA‐PRESS TE) (Mullins et al., 2008), or using a multi‐TE PRESS sequence to generate a TE‐averaged spectrum with a well‐resolved Glu signal (Hurd et al., 2004). Importantly, our recent publication demonstrated that Glu can also be measured from the sum spectrum of the MEGA‐PRESS GABA data (sum = ON + OFF), resulting in negligible overlap with glutamine and a strong association with the TE‐averaged Glu (Saleh et al., 2023).

In this study, we employ the MM‐suppressed MEGA‐PRESS sequence to study GABA (with reduced co‐edited MM contamination) and Glu from the sum spectrum in the temporal cortices (covering the STG) of children with ASD, followed by correlations of these chemicals with ASD severity ratings. Although many MRS studies, including our previous work (Gaetz et al., 2014), focused on the left hemisphere due to its (albeit weak) association with the receptive language in the ASD compared with the TD population (Bigler et al., 2007), a previous brain volumetric study revealed a significant increase in the right STG volume of the ASD population (Jou et al., 2010), suggesting that the right hemisphere might also be implicated in autism. Considering these findings implicating the right and left STG in autism, this study aims to investigate brain GABA and Glu levels in both hemispheres. Where possible, we seek to replicate the conditions of prior reports (with smaller N) using a subset of our acquired data (and permutation methods) to establish the probability of consistency and to implicate methodological limitations of prior reports for any residual inconsistency. Due to discrepancies in the literature—including our own previous work with a small sample size in the table—we took a “discovery” approach, free from unsubstantiated hypotheses and bias to GABA and Glu concentrations. Our sample size provides a unique opportunity to determine the existence of any alteration, the direction of these changes, and association with clinical traits, and in doing so, address discrepancies in the literature.

## METHODS

The Children's Hospital of Philadelphia Institutional Review Board approved the study and consent forms. Parents or guardians gave written consent, and children also gave verbal assent to participate.

### Participants

Children were enrolled in the study, underwent diagnostic assessment, and subdivided into ASD or typically developing (TD) groups through the same channels reported in the previous publication (Roberts et al., 2010). Briefly, all children screened for inclusion in the ASD sample had a prior ASD diagnosis made by an expert clinician, typically a developmental pediatrician at the Children's Hospital of Philadelphia. Furthermore, clinical and diagnostic testing was performed during their in‐person visit to confirm the referral ASD diagnosis, administer neuropsychological tests, and ensure that the TD children met the study criteria provided in a previous report (Roberts et al., 2010). 86 children met the study eligibility requirements and completed the study procedures. 56 participants, ages 7.5 to 17.9 years (mean ± SD: 12.2 ± 2.6 years; 5/51 F:M), had confirmed ASD diagnosis using standard diagnostic tools, including the Autism Diagnostic Observation Schedule‐2nd Edition (ADOS‐2) (Lord et al., 2012) and parent report on the Social Communication Questionnaire (SCQ) (Rutter et al., 2003). A second group of participants comprised 30 TD, ages 8.1 to 17.7 years (12.1 ± 2.9 years; 2/28 F:M), with no history of developmental disability or neurological disorder. Parent reports on the Social Responsiveness Scale‐2nd Edition (SRS‐2) (Constantino & Gruber, 2012) provided dimensional ratings of ASD‐associated levels of socialization and repetitive/restricted behavior traits. A measure of full‐scale IQ (FSIQ) was obtained for all participants. The specific measure of IQ utilized varied depending on the group and included the Differential Ability Scale—Second Edition (DAS‐II) (Elliott, 2007) and Wechsler Intelligence Scale for Children—5th Edition (WISC‐V) (Wechsler, 2014). All assessments were performed by a licensed child psychologist with expertise in autism (LB). A small portion of the data from some of these participants has been partially reported in prior studies focused on multimodal modeling of electrophysiological responses in ASD.

### Acquisition

**FIGURE 1:**
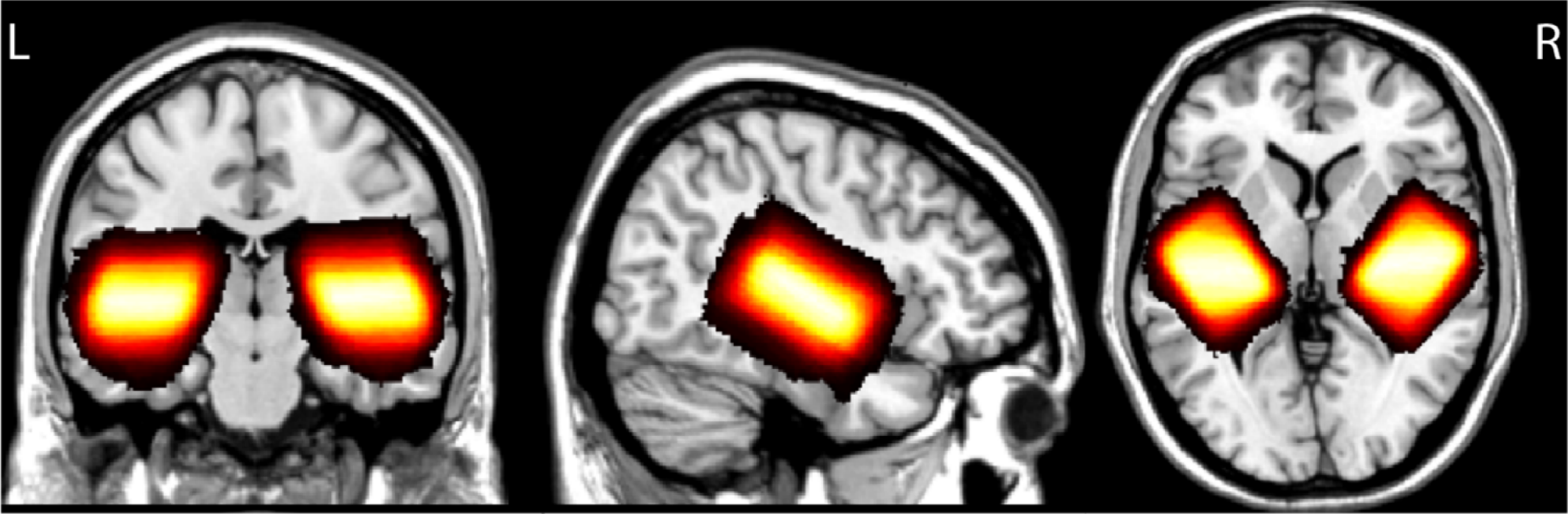
Edited MRS voxel locations in the left and right auditory cortices. Every participant's voxel mask from both regions (separately) were transformed from their native space to MNI space and overlaid onto the Colin Holmes (CH) template. Light colors (yellow color) indicate areas of greater overlap across participants. The transformation was done for visual illustration only (not for metabolite concentration estimations).

Magnetic resonance imaging and spectroscopy (MRI/MRS) experiments were performed on a 3 T Verio MRI scanner (Siemens, Erlangen, Germany). A whole‐brain T1‐weighted 1‐mm isotropic voxel resolution structural image (field of view = 256 × 256 × 176 mm and matrix 256 × 256 × 176) was acquired in every participant to guide voxel placements in the left and right auditory cortices (Figure 1). Briefly, the voxel on each side was first aligned to the mid‐temporal lobe with the long aspect of the cuboid aligned such that the top of the voxel contains the superior temporal gyrus (STG). Adjustments were made to reduce the inclusion of the lateral ventricle. The vendor‐provided MEGA‐PRESS works‐in‐progress 529 sequence (Mescher et al., 1998) was used to acquire MM‐suppressed GABA in both cortices with editing pulses applied at 1.9 ppm (edit ON) and 1.5 ppm (edit OFF) in an interleaved fashion, symmetric about 1.7 ppm. The acquisition parameters were TR/TE 1500/80 ms, 25.6 ms editing pulses (35 Hz set on the scanner), 1056 datapoints, 2.4 kHz spectral width, 30 mm (right–left) × 40 mm (anterior–posterior) × 20 mm (foot‐head) voxel size; 128 transient pairs; and WET water suppression. Outer‐volume suppression pulses were applied to saturate spins outside the volume of interest and reduce signals from outside the voxels. The total scan time per hemisphere was about 7 minutes. Finally, water‐unsuppressed data were acquired from both cortical ROIs (for water scaling quantification) with the same parameters, except for 32 transients and TR 6000 ms.

### Processing

In vivo MEGA‐PRESS data were processed in Osprey (version 2.5.0) (Oeltzschner et al., 2020). The 3.02‐ppm total creatine (tCr = creatine + phosphocreatine) signal was used to estimate magnetic field (B_0_) drift in the in vivo data before frequency‐and‐phase correction (FPC). Within every MRS dataset, any transients with a B_0_ drift exceeding 5 Hz from the first transient (reference transient) were removed, and the number of remaining transients was recorded. Using these recorded numbers, the overall mean and standard deviation were calculated (mean ± SD = 215.02 ± 56.14 transients). Any MRS dataset with the remaining transients (i.e., those surviving the 5‐Hz threshold) two standard deviations below the mean (215.02–2*56.14 = ~102) was removed and excluded from further analysis. Finally, FPC (Mikkelsen et al., 2018) was performed on the remaining transients of every surviving MRS dataset separately. The fully processed sub‐spectra were combined to generate the difference or GABA‐edited (ON – OFF) and sum (ON + OFF) spectra. The residual water signal was removed using an HSVD filter (Barkhuijsen et al., 1969).

### Modeling

The in vivo GABA‐edited (difference) and sum spectra were modeled using LCModel (6.3–1 N, http://s-provencher.com/lcmodel.shtml). 2D localized density‐matrix simulations of the ascorbate, aspartate, creatine, ethanolamine, GABA, glycerophosphocholine (GPC), glutathione, glutamine, Glu, myoinositol, N‐acetylaspartate, N‐acetyl‐aspartyl‐Glu, phosphocholine, phosphocreatine, phosphorylethanolamine, scyllo‐inositol, and taurine spin systems were performed in MRSCloud (Hui et al., 2022) following the MEGA‐PRESS experiment to generate LCModel basis sets. The following were the simulation parameters: ideal excitation pulse; TE 80 ms; 101 × 101 two‐dimensional spatial array in the refocusing dimensions; 8192 complex data points; 4 kHz spectral width; simulated linewidth of 2 Hz. The water‐scaled concentrations of GABA were estimated from the difference spectra, modeled between 1.88 and 4 ppm with the following parameters in the LCModel control file: “*sptype = mega‐press‐3*”; “*nobase = F*”; baseline knot spacing = 0.6; and “*dows = T*.” The water‐scaled Glu and tCr concentrations were estimated from the sum spectrum (Saleh et al., 2023), modeled between 1.8 and 4 ppm. The T1‐weighted images were segmented to calculate gray matter (GM), white matter (WM), and CSF voxel tissue fractions. GABA, Glu, and tCr concentrations were corrected for partial volume and relaxation effects in each voxel using the method described by Gasparovic and colleagues (equation in *OspreyQuantify* [Gasparovic et al., 2018]). The GABA/tCr (i.e., water‐scaled GABA from the difference spectrum divided by the water‐scaled tCr from the sum spectrum), Glu/tCr, and GABA/Glu ratios were estimated from the water‐scaled concentrations and only corrected for relaxation effects (T_1_ and T_2_). Our main findings are from the water‐scaled concentrations—the “relative to tCr” measurements are supplementary (and used for comparison with the prior literature that used tCr as the reference for quantification).

### Analysis

The averaged difference and sum spectra from both voxels were visually assessed, and those with spurious signals (from outside the voxels) or baseline distortions were removed from further analysis. From the remaining datasets, individual spectra and quantitative parameters—the Cramer Rao Lower Bounds (CRLB), SNR, frequency drift (Hz), and linewidth (Hz)—were recorded. Statistical analyses were conducted using R software (version 4.4.0, http://www.r-project.org). Unless otherwise stated, values are presented as mean or mean ± SD.

One‐way analyses of covariance (ANCOVA) were performed for every region to evaluate the effects of group status (GS: ASD or TD), age and sex were covariates, and the interaction of group‐by‐age on brain chemicals (GABA, GABA/tCr, Glu, Glu/tCr, GABA/Glu, and tCr separately). Since the age range in both groups was wide (~10 years) and potentially influenced metabolite concentrations, age was included as a covariate in our group analyses (Port et al., 2017; Saleh et al., 2020). If the group‐by‐age interaction was a trend (*p* ≤ 0.1) or significant, post‐hoc correlation analyses were performed using Pearson (r) or Spearman correlations (ρ), depending on the residual distribution. Otherwise, the model was repeated without the interaction terms. A *p*‐value ≤0.05 was considered statistically significant. Cohen's d was also calculated to determine the effect size of the chemical difference between the two groups (ASD and TD). Cohen's |d| value ranging between 0 and less than or equal to 0.2 represented a negligible effect, greater than 0.2 and less than or equal to 0.5 represented a small effect, greater than 0.5 and less than or equal to 0.8 represented a medium effect, and greater than 0.8 represent a large effect.

Separate correlation analyses determined the association between each brain chemical level and the Social Responsiveness Scale (SRS). To reduce the overlap in the measures of SRS of the ASD group with those of the TD, we calculated the 98% confidence interval (98% CI: 44–107) in the ASD SRS values. We used the lower bound of the CI as the inclusion threshold for the ASD scores. This threshold excluded a single ASD participant with an SRS score of 43 (below the lower bound of the CI) before the correlation analysis. Further outliers were identified using Cook's distance estimates, excluding values higher than 4/*n*, where *n* is the sample size. A *p*‐value of ≤0.025 (2 chemicals: 0.05/2) was considered statistically significant after applying the Bonferroni method to correct multiple comparison analyses for SRS correlations with two chemicals.

Finally, we performed permutation analyses (1000 iterations) to calculate the probabilities of reproducing the findings reported in the previous studies using their respective ASD sample sizes (N_ASD_) and our TD (N_TD_ = 30). For each iteration, the permutation function *slice_sample* (from the *dplyr* library in R) was used to randomly select N_ASD_ from our dataset, followed by a 2‐tailed t‐test (or Wilcoxon rank sum test if not normally distributed) to determine the difference between the two groups (N_ASD_ vs. our N_TD_). A *p*‐value ≤0.05 was considered statistically significant.

## RESULTS

### Demographics

Full participant characteristics are reported in Table 2. TD and ASD participants had similar age and sex distributions. In agreement with the expected clinical/behavioral phenotype, the ASD group had significantly higher SRS, lower FSIQ, and higher ADOS scores than the TD group. The ASD and TD groups had similar tissue compositions of MRS voxels in both hemispheres, except for a small CSF difference in the right hemisphere of the ASD group (~1% higher).

**TABLE 2:** Participant characteristics and voxel‐tissue composition.

	ASD (*N* = 56)	TD (*N* = 30)	*p*‐value
Sex (M/F)	51/5	28/2	1.00^a^
Age, years	12.15 ± 2.65	12.06 ± 2.94	0.89^b^
Range, years	7.48–17.93	8.12–17.70	
SRS (T scores)^c^	73.5 ± 12.4	42.5 ± 4.09	<0.001^b^
Full scale IQ (standard scores)^d^	100.6 ± 18.2	114.4 ± 14.3	<0.001^b^
ADOS (CSS scores)^e^	6.84 ± 2.01	1.29 ± 0.85	<0.001^b^
Auditory cortex LH (%)			
GM	54.37 ± 6.69	53.38 ± 5.41	0.47^b^
WM	41.50 ± 7.12	43.03 ± 4.96	0.25^b^
CSF	4.13 ± 1.52	3.58 ± 1.71	0.16^b^
Auditory cortex RH (%)			
GM	54.33 ± 5.41	53.54 ± 5.1	0.51^b^
WM	41.28 ± 5.87	42.95 ± 5.35	0.20^b^
CSF	4.39 ± 1.75	3.51 ± 1.23	0.01^b^

*Note*: Data are presented as *N* or mean ± SD.

Abbreviations: ADOS, autism diagnostic observation schedule; CSF, cerebrospinal fluid; CSS, calibrated severity score; GM, gray matter; SRS, social responsiveness scale; WM, white matter.

^a^ Chi‐square test.

^b^ *t*‐test or non‐parametric *t*‐test.

^c^ SRS measured from 55 ASD and all TD;

^d^ Full Scale IQ measured from 53 ASD and all TD;

^e^ ADOS measured from all ASD and 28 TD;

### Data quality

**FIGURE 2:**
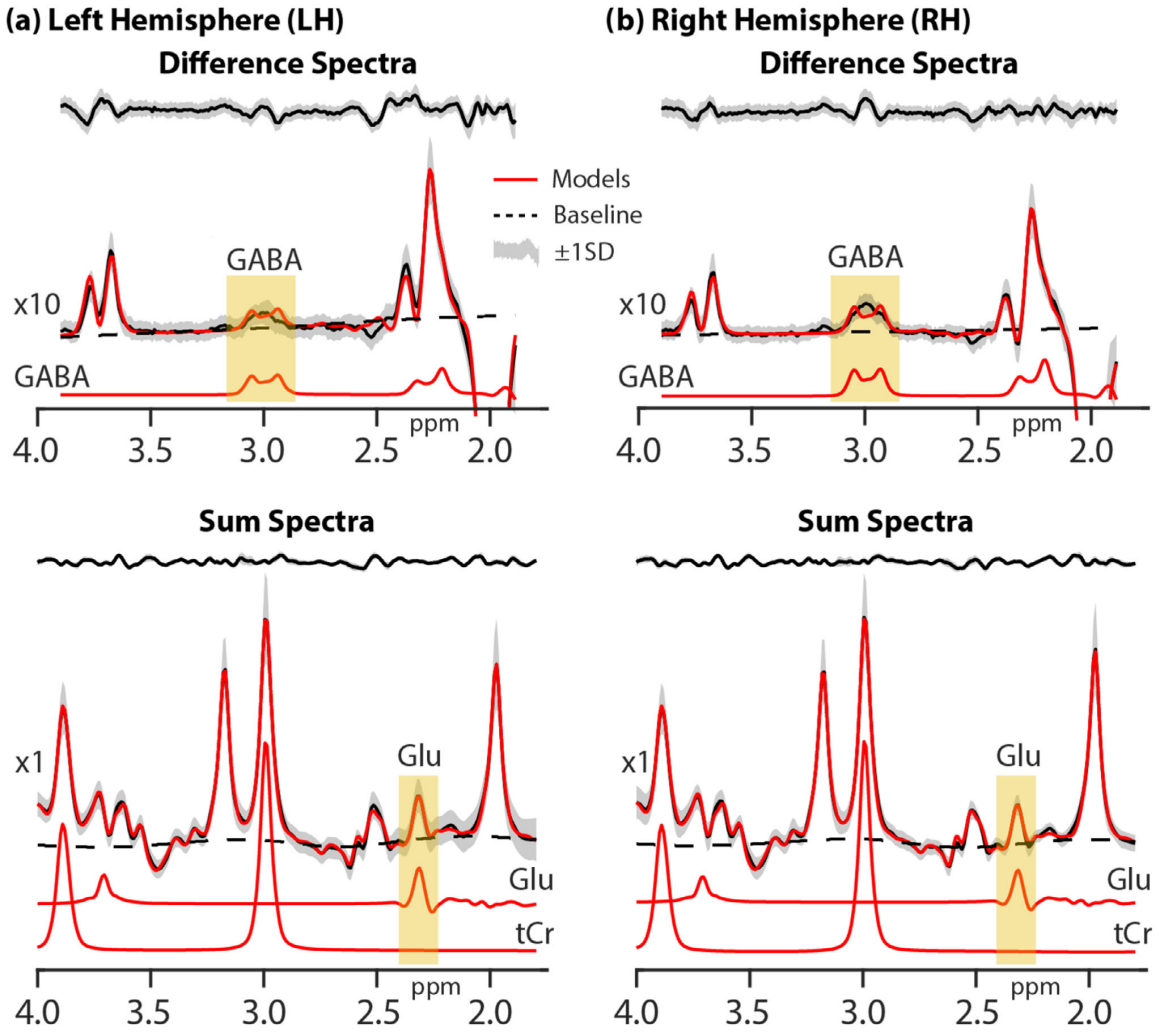
In vivo difference (top) and sum spectra (bottom) acquired from the (a) left hemisphere (LH) and (b) right hemisphere (RH). The black continuous line within each spectrum is the overall average spectrum (ASD and TD combined) overlaid with ±1SD of the combined data (gray) and models (red). The difference spectra were scaled by a factor of 10 for visualization purposes only. ASD, autism spectrum disorder; LH, left hemisphere; RH, right hemisphere; TD, typically developing.

In total, data from 18 hemispheric ROIs (ASD/TD: 9/0 in the LH and 8/1 in the RH) were removed before the final group analysis due to the data not meeting the qualitative and quantitative standards (minimum number of surviving transients, visual inspection, etc.). 8 ASD participants had no evaluable datasets after outlier rejection. After removal, the average difference and sum spectra from each hemisphere overlaid with the corresponding variation in the spectra (±1SD in gray) and averaged linear combination models (in red) were generated (Figure 2). The GABA signal is detected at 3 ppm in the difference spectra, and the Glu signal is co‐detected at 2.34 ppm in the sum spectra. The qualitative parameters are presented in Table S1, and individual spectra from each region and group are shown in Figures S1 and S2.

### Left hemisphere

**FIGURE 3:**
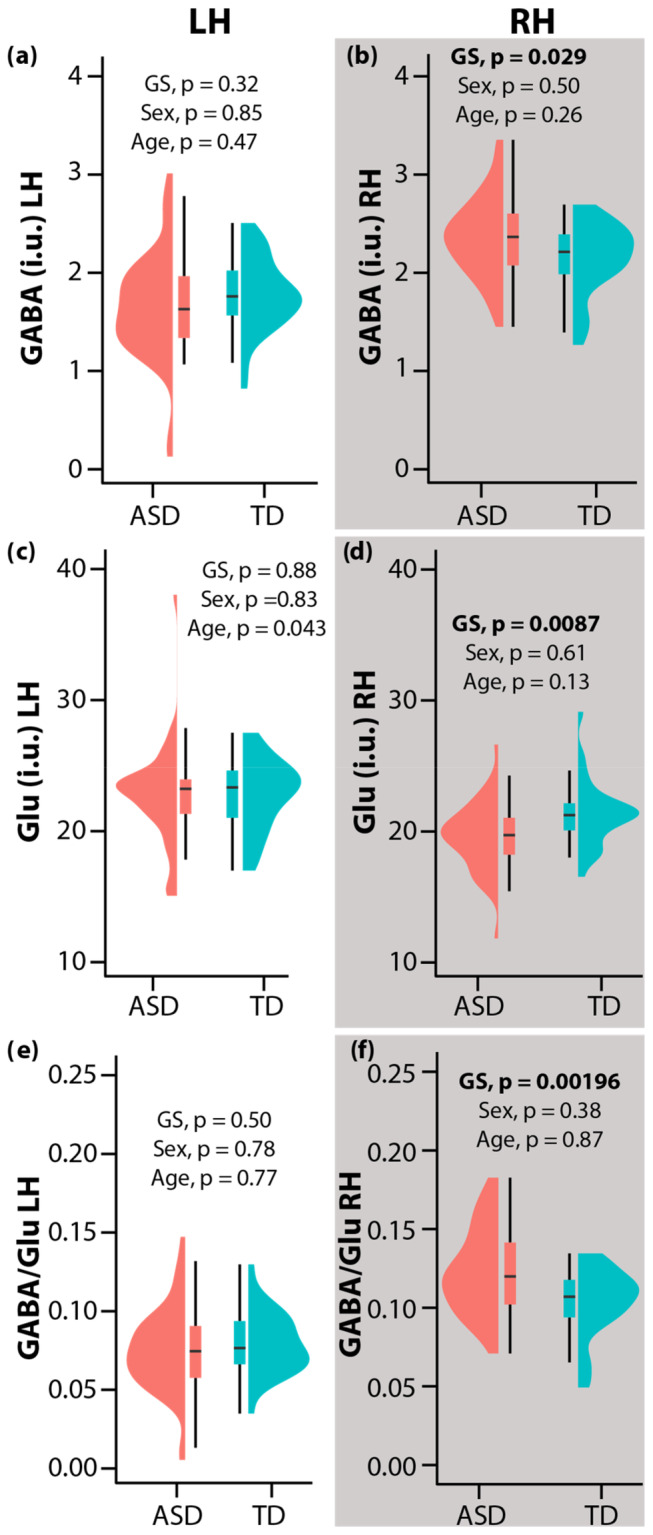
Raincloud plots of GABA (a and b), Glu (c and d), and GABA/Glu (e and f) in the left (left) and right (right) hemispheres. The statistical results are from running ANCOVA for every metabolite (separately) in each region with age and sex as covariates. Only GABA, Glu, and GABA/Glu in the right hemisphere are significantly different between the groups (*p* < 0.05). ASD, autism spectrum disorder; GS, group status; i.u., institutional units; LH, left hemisphere; RH, right hemisphere; TD, typically developing; *p*‐values for the GS, sex, and age effects are from ANCOVA. Right hemispheric results are highlighted to depict statistically significant group differences.

Figure 3 and Figure S3 show group differences relative to water and tCr in both hemispheres, respectively. In the left hemisphere, neither GABA (percentage difference in ASD means: –6.6%; F (1, 71) = 1.007, *p* = 0.32; Cohen's |d| = 0.24, a small effect), Glu (−0.5%; F (1, 71) = 0.021, *p* = 0.88; Cohen's |d| = 0.03, a negligible effect), nor GABA/Glu (−5.1%; F (1, 71) = 0.470, *p* = 0.50; Cohen's |d| = 0.16, a negligible effect) showed significant group differences. There was a significant age effect when examining Glu levels (decreasing with age, F (1, 71) = 4.236, *p* = 0.043).

Non‐significant group differences were found in GABA/tCr (−8.2%; F (1, 71) = 1.750, *p* = 0.19; Cohen's |d| = 0.32, a small effect), Glu/tCr (−1.1%; F (1, 71) = 0.1803, *p* = 0.67; Cohen's |d| = 0.1, a negligible effect), and tCr (0.6%; F (1, 71) = 0.043, *p* = 0.84; Cohen's |d| = 0.05, a negligible effect), but a significant sex effect was found in Glu/tCr levels (females>males, F (1, 71) = 5.400, *p* = 0.023).

Neither GABA (ASD/TD: r = 0.05, *p* = 0.764/r = −0.253, *p* = 0.195), Glu (ASD/TD: r = −0.09, *p* = 0.560/r = 0.201/*p* = 0.296), nor GABA/Glu (ASD/TD: r = −0.023, *p* = 0.888/r = −0.312, *p* = 0.106), showed a significant correlation with the SRS scores in either of the groups.

### Right hemisphere

In the right hemisphere, the ASD group had significantly higher GABA (percentage difference in ASD means: **9.8%**; **F(1, 68)** = **4.951**, ***p*** = **0.029**; Cohen's |d| = 0.53, a medium effect), lower Glu (**−7.9%**; **F(1, 68)** = **7.289**, ***p*** = **0.0087**; Cohen's |d| = 0.65, a medium effect), and higher GABA/Glu (**19.2%**; **F(1, 68)** = **10.380**, ***p*** = **0.00196**; Cohen's |d| = 0.78, a medium effect).

Similar to the above results were observed in Glu/tCr (−11.0%; F (1, 68) = 17.103, *p* < 0.01; Cohen's |d| = 0.96, a large effect), but GABA/tCr was non‐significantly higher (6.0%, F (1, 68) = 1.868, *p* = 0.18; Cohen's |d| = 0.33, a small effect). Although there was no group difference in tCr (3.4%, F (1, 67) = 2.152, *p* = 0.15; Cohen's |d| = 0.34, a small effect), a strong interaction between group status (F (1, 67) = 4.691, *p* = 0.034) and age was observed, driven by opposite, significant age‐related changes in TD (ρ = 0.495, *p* = 0.006) and non‐significant age‐related changes in ASD (r = −0.104, *p* = 0.51).

**FIGURE 4:**
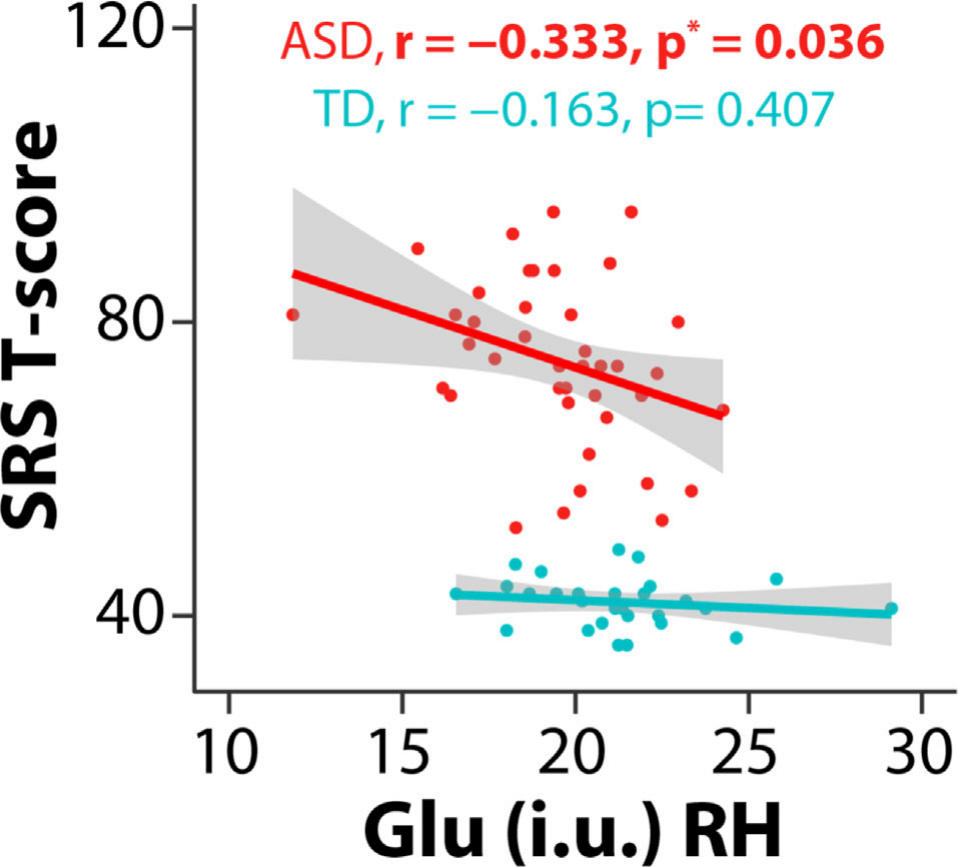
SRS correlations with Glu in the ASD and TD groups separately. ASD, autism spectrum disorder; i.u., institutional units; RH, right hemisphere; SRS, social responsiveness scale; TD, typically developing; p*: *p*‐value did not survive multiple comparison.

GABA in the TD showed a trend toward correlation with SRS scores (ASD/TD: r = 0.062, *p* = 0.711/r = −0.349, *p* = 0.074), with increased GABA associated with lower SRS scores. Of note, Glu in ASD showed a significant negative correlation with SRS (ASD/TD: **r = −0.333, *p* = 0.036**/r = −0.163, *p* = 0.407; Figure 4) but did not survive the multiple comparison correction. GABA/Glu (ASD/TD: r = 0.249, *p* = 0.132/r = −0.071, *p* = 0.721) did not yield a significant association with the SRS scores in either of the groups.

### Permutations

The results of the permutation analyses are in Table 3. Following the reported sample size (N_ASD_), most of the studies agree with the direction of changes (Edmondson et al., 2020; Gaetz et al., 2014; Joshi et al., 2013; Kolodny et al., 2020; Port et al., 2017; Rojas et al., 2014), one study is more likely to agree than disagree (DeVito et al., 2007), and only one study (Brown et al., 2013) disagreed with our direction.

**TABLE 3:** 1000 permutations to calculate the probabilities of reproducing (or opposing) prior findings using the study ASD sample size (N_ASD_) and our TD (N_TD_ = 30).

Reference	N_ASD_ reported findings	Glu or Glx		GABA		
		ASD < TD	ASD > TD	ASD < TD	ASD > TD	Directional concordance with present study
Present study	56 ASD Signif. Glu ASD < TD in children RH Signif. GABA ASD > TD in children RH (all institutional units)					
DeVito et al., 2007	26 Trend Glu ASD < TD in children LH	LH 0% Signif 0.3% Trend 59.2% NS^a^	LH 0% Signif 0% Trend 40.5% NS			More likely to agree (59.5%) than disagree (40.55)
Brown et al., 2013	13 Signif. Glu ASD > TD in adults LH and RH	LH 0.5% Signif 1.9% Trend 53.0% NS^a^ RH 48.2% Signif^a^ 16.6% Trend 35.1% NS	LH 0.0% Signif 0.1% Trend 44.5% NS RH 0.0% Signif 0.0% Trend 0.1% NS			Disagree (esp. in RH), but note age‐difference in population as well as small sample and other methodological differences
Joshi et al., 2013	7 Trend Glu ASD < TD in children RH	RH 27.2% Signif^a^ 16.0% Trend 53.8% NS	RH 0.0% Signif 0.0% Trend 3.0% NS			Agree in direction, with significance in 27.2% of permutations (but note very small N)
Rojas et al., 2014	17 Signif. GABA ASD < TD in children LH			LH 5.3% Signif 7.2% Trend 82.0% NS^a^	LH 0.0% Signif 0.0% Trend 5.5% NS	Agree in direction (94.5%) although might only attain significance in 5.3% of our sample permutations
Gaetz et al., 2014	17 Signif. GABA ASD < TD in children LH			LH 5.3% Signif 7.2% Trend 82.0% NS^a^	LH 0.0% Signif 0.0% Trend 5.5% NS	Agree in direction (94.5%) although might only attain significance in 5.3% of our sample permutations
Port et al., 2017	16 Signif. GABA ASD < TD in children LH			LH 4.7% Signif 7.4% Trend 81.3% NS^a^	LH 0.0% Signif 0.0% Trend 6.6% NS	Agree in direction (93.4%) although might only attain significance in 4.7% of our sample permutations
Kolodny et al., 2020	31 NS for both chemicals in adults LH (all institutional units)	LH 0.0% Signif 0.1% Trend 60.5% NS^a^	LH 0.0% Signif 0.0% Trend 39.4% NS	LH 2.2% Signif 4.9% Trend 91.2% NS^a^	LH 0.0% Signif 0.1% Trend 1.7% NS	Glu: agree NS, with slight bias toward decrease in ASD GABA: agree NS although might attain significance in 2.2% of our sample permutations
Edmondson et al., 2020	21 NS for GABA Signif. Glx ASD < TD in children RH (all institutional units)	RH 65.7% Signif^a^ 17.7% Trend 16.6% NS	RH 0.0% Signif 0.0% Trend 0.0% NS	RH 0.0% Signif 0.0% Trend 0.0% NS	RH 40.0% Signif^a^ 21.6% Trend 38.4% NS	Glx: Agree in direction (100%) and significant ~66% of our permutations GABA: Agree in direction (100%) although might attain significance in 40% of our sample permutations

*Note*: Prior literature reports used earlier acquisition and analysis methods as well as smaller population samples. We examined through permutation analysis in our larger sample, the probability of reproducing these prior findings—supporting the argument of small sample bias, vs. opposing them, supporting methodological differences. While both factors contribute, it is clear that larger samples and state‐of‐the‐art methods, as reported in our study resolve some of the inconsistencies in prior reports.

Abbreviations: ASD, autism spectrum disorder; LH, left hemisphere; NS, not significant (*p* > 0.1); RH, right hemisphere; Signif, statistically significant (*p* ≤ 0.05); tCr, total creatine; TD, typically developing; Trend, 0.05 < *p* ≤ 0.1.

^a^ The outcome matching the results of the present study.

## DISCUSSION

We evaluated brain abnormalities in a moderately large sample of autistic children using an MM‐suppressed spectrally edited MRS technique to measure GABA and Glu concentration in the auditory cortices. ASD participants show significant group differences compared to TD controls in both GABA and Glu levels, but only in the right auditory cortex: the ASD group has higher GABA concentration; lower Glu concentration; and higher GABA/Glu in the right hemisphere. Our RH findings of GABA disagreed with the previous study (Edmondson et al., 2020). However, our RH Glu findings agreed (Edmondson et al., 2020; Joshi et al., 2013) and disagreed (Brown et al., 2013) with the reported findings. The LH revealed no significant changes in chemical levels, similar to the previously reported results (Edmondson et al., 2020; Joshi et al., 2013; Kolodny et al., 2020), but disagreed with others (Brown et al., 2013; Gaetz et al., 2014; Port et al., 2017). The brain asymmetry found here was also found in a previous non‐MRS study (Jou et al., 2010), where volumetric differences were found only in the right STG of the ASD population (similar age range to ours). These asymmetric findings suggest the importance of investigating both hemispheres in ASD to characterize brain abnormalities better.

Elevated GABA concentrations in the right hemisphere of children with ASD suggest abnormal GABAergic neurotransmission. GABA is linked with important functions in the developing brain, including myelination and synaptic pruning, allowing the development of new cognitive abilities, such as comprehending language (Williamson & Lyons, 2018). Abnormal levels of GABA found in this study may have originated from early developmental differences resulting in the disruption of myelination in young children (Chen et al., 2022), and suggest that it may continue to disrupt during the later stages of development (Hamilton et al., 2017).

The main finding of lower right hemispheric Glu levels in individuals with ASD suggests abnormal glutamatergic neurotransmission. Glu plays an important role in the neurodevelopmental process, including neuronal migration, to allow proper nervous system functioning, such as language comprehension (Li et al., 2020; Meldrum, 2000). Alterations in the Glu levels could result from early developmental differences (Chen et al., 2022), and considering that the brain continues to mature till the age of ~25 years, abnormally lower Glu levels may impact further the nervous system development in our cohort (Sowell et al., 2003). We also found lower Glu levels associated with more severe SRS scores, suggesting that Glu plays a role in ASD symptomatology, including problems with social interaction and demonstrating social isolation. Again, the hemispheric specificity of the current observation has implications for functional specialization during brain development.

The abnormal levels of GABA and Glu resulted in higher GABA/Glu in ASD, supporting an excitatory‐inhibitory (E/I) imbalance (Rubenstein & Merzenich, 2003). The E/I imbalance is suggested to be among the underlying mechanisms in ASD responsible for information processing (Culotta & Penzes, 2020). E/I imbalance involves an intricate combination of brain chemicals and might be present regionally (hemispheric/regional differences). In the broadest sense, departures from “balance” can occur in either direction or represent an “atypicality” (Nisar et al., 2022). Decreased Glu, in itself, would not be directly consistent with the association with epilepsy (the widely adopted basis for the E/I imbalance hypothesis in ASD), except that it must be remembered that GABA, glutamine, and Glu are elements of a complicated, intricate, balance, and our spatial resolution inherently averages over different tissue types. Given that the E/I imbalance in this study appears due to aberrations in GABA and Glu levels, novel treatments targeting both GABAergic and glutamatergic systems may improve the pathophysiology of ASD.

MRS studies in ASD span over multiple brain regions (Brix et al., 2015; Dionísio et al., 2024; Du et al., 2023; Puts et al., 2017)—including the anterior cingulate cortex, motor cortex, and thalamus—revealing regional variation in GABA and Glu (or Glx, Glu + glutamine). Since the current study focused on the auditory cortex, permutation analyses were conducted to determine the probability of reproducing prior findings in the same region (Table 3). The analysis revealed hemispheric and directional consistency with most of the studies. The probability of reproducing the GABA's direction had a smaller range (~93%–100%) than the Glu's direction (~60%–100%). The wide range in probabilities for Glu could be attributed to the methodological differences between our studies in measuring the chemical. For example, Devito and colleagues performed spectroscopy acquisitions at a longer TE (135 ms vs. our at 80 ms), adding more T_2_ relaxation effects in their Glu results (DeVito et al., 2007). The main discrepancy between the present study and the prior literature is with the RH findings of Brown et al., who reported *elevated* Glx in ASD (Brown et al., 2013). Notably, their study population was entirely adults (~37 years), and their methodology differed. The authors measured Glx in adults using the standard unedited MRS and found higher Glx in the ASD group in both hemispheres (Brown et al., 2013). The age difference between their study and ours is more than 20 years, during which the ASD group might have undergone brain maturation and alteration in the trajectory of brain neurotransmitter levels (Brown et al., 2013). Taken together, these prior studies have added significant knowledge to the literature about brain chemistry in the context of ASD, providing the impetus for our study with a larger sample size to address disagreements in the literature.

We analyzed our data with both normalization methods – relative to water and relative to tCr—to examine the group differences in GABA and Glu levels. Since the ratios of GM to WM concentrations of GABA, Glu, and tCr are known to be dissimilar (Saleh et al., 2017), our measurements relative to water were corrected for tissue fractions. The pattern of results did not change when scaling to tCr, except for non‐significant GABA/tCr in the right hemisphere. The breakdown of voxel composition shows that ASD and TD did not substantially differ in GM and WM fractions, removing a potential tissue confound in our metabolite relative to tCr analyses.

Several studies show stability in tCr across clinical conditions and use it as a reference to report metabolite levels (Rackayova et al., 2017). Although we found no group difference in tCr, the TD (but not ASD) group's age effect might have influenced age‐covaried GABA/tCr group‐level differences. The statistical results of the reference, tCr (including age effect), should be considered when interpreting the metabolite ratios to tCr.

While a portion of the data from some of these participants has been *partially* reported in prior studies focused on multimodal modeling of electrophysiological responses in ASD (ASD/TD *N* = 52/30 using the GABA/Cr levels only) (Berman et al., 2023; Roberts et al., 2020), notable methodological advancements have been applied in the present study, including use of linear combination modeling, use of water scaling, consideration of not only GABA but importantly, Glu as well as tissue composition/relaxation correction. Broad consistency with these prior analyses was preserved (non‐significant overall group effect on GABA, with left hemispheric decrease and right hemisphere increase), while the present study offered an extension to identify more pronounced effects on Glu (significant right hemispheric decrease in ASD) and, indeed, on estimates of E/I balance itself (significant right hemispheric increase in GABA/Glu ratio) as well as identification of a novel clinical correlate of MR spectroscopic findings (association between SRS and right hemispheric Glu levels).

## LIMITATIONS

While the primary focus is the superior temporal gyrus as it houses auditory processing and conduit to the receptive language areas of the posterior superior temporal gyrus (STG), the relative insensitivity of the single voxel edited MRS technique and the relatively low signal amplitudes of visible metabolites necessitates relatively large voxels (~30 mL), encompassing STG/middle temporal gyrus areas anatomically (capturing the majority of receptive language fields identified during invasive speech mapping, for example) (Ojemann, 1991). Employing a MEGA‐edited scheme in other localization techniques with better slice profiles and lower chemical shift displacement artifacts (Garwood & DelaBarre, 2001; Saleh et al., 2018) might enable smaller voxels and allow region‐specific analyses of GABA and Glu.

Another limitation is the difference in general cognitive abilities between the two groups as indexed by IQ. Even though both the ASD and TD groups are in the normal range of IQ, their mean values are different by one standard deviation. Consequently, our interpretation of the metabolite concentration differences could also be attributed, in part, to general cognitive differences and autism diagnosis. However, as shown in the supplementary data (Figure S4), group differences in RH Glu persist across the full range of IQ scores, and RH Glu is not associated with IQ in either group.

We employed the MM‐suppressed technique to measure GABA in the brain. This sequence demands high fidelity in applying the editing pulses symmetrically, about 1.7 ppm, to allow proper suppression of MM. We assessed our datasets qualitatively and quantitatively to reduce the inclusion of poor‐quality data and increase the accuracy of measurements. We removed any transient with excessive frequency drift (beyond 5 Hz from the first transient) caused by subject motion or B_0_ drift, datasets with insufficient transients to avoid lower SNR in the combined data, and visually assessed the combined spectra for any artifacts or misalignments. The literature has suggested different methods for removing unwanted transients (“*bad data*”), including transients affected by instabilities (e.g., motion) using unlikeness metrics (Near et al., 2011; Near et al., 2013; Saleh, Alhamud, et al., 2016), visual inspection to determine the polarity of the GABA signal (affected by a varying range of drifts) (Edden et al., 2016), or using a prospective navigator in identifying motion‐corrupted transients during post‐processing (Bhattacharyya et al., 2007). Our primary assessment of unwanted datasets considered the uncertainties in estimating the water (i.e., ranging between 4.65 and 4.70 ppm between different scanner vendors [Saleh et al., 2019]) and editing frequencies (i.e., a value between 1.88 and 1.91 ppm for GABA [Gonenc et al., 2010; Govind et al., 2015; Rothman et al., 1993]) during the MRS scan setup (±2–3 Hz) and a small error in estimating the Cr frequency during post‐processing (±1–2 Hz.). From these observations, ±5 Hz was used as a threshold for the transient inclusion, which reduced improperly edited GABA and MM contamination in our processed datasets. We could narrow the threshold (e.g., ±2 Hz to reduce MM contamination further) but at the cost of fewer surviving transients for frequency‐and‐phase correction, possibly resulting in misalignment of transients, increased spectral artifacts, and reduced measurement accuracy (Near et al., 2015). In future studies, our approach, coupled with the prospective frequency‐and‐motion‐correction navigators, can further improve editing, lower the number of rejections, and enhance data quality (Saleh, Alhamud, et al., 2016; Saleh, Near, et al., 2016).

This study had a small (<1%) but significant CSF difference between the two groups. Our previous reproducibility work demonstrated high repeatability of the voxel placement procedures in the region encompassing the auditory cortex (Gaetz et al., 2014). Despite the careful attempts to place voxels in similar locations, slight variations in positioning necessarily occur, resulting in small variations between the subjects, such as the CSF difference found in this study. To account for these differences, measurements were corrected for tissue fractions, including CSF, during the quantification steps.

Our study is limited to two brain regions and two chemicals, whereas other cortices (Gaetz et al., 2014; Harada et al., 2011) and chemicals are implicated in ASD (e.g., glutathione [Frustaci et al., 2012]). Employing MRSI methods capable of studying multiple brain regions in combination with multiplex editing would comprehensively characterize brain chemistry in individuals with ASD (Chan et al., 2019). Furthermore, our study's cross‐sectional nature only sheds light on chemical changes (or lack thereof) at a single time point. Longitudinal follow‐up of our cohort would provide a unique opportunity to understand the trajectory of the brain chemicals in ASD.

## CONCLUSION

We examined the auditory cortices of individuals with ASD using edited MRS. We found right hemispheric differences: higher GABA concentration, lower Glu concentration, and higher GABA/Glu ratios than their typically developing counterparts. Alterations in GABA and Glu levels suggest impaired neurotransmitter regulation and E/I imbalance. Previous literature reports in similar populations (albeit with smaller samples) have shown mixed findings; we believe this larger‐scale study, combined with state‐of‐the‐art acquisition and analysis methods, might resolve prior published ambiguities. Furthermore, since literature displays differing results between adults and children, future longitudinal studies would provide important insight into the changes in neurotransmitter balance over development and transition into adulthood.

## ETHICS STATEMENT

The study was approved by the CHOP Institutional Review Board and all participants' families gave written informed consent. As indicated by institutional policy, where competent to do so, children additionally gave verbal assent, in accordance with the principles of the Declaration of Helsinki.

## Supporting information


**Data S1:** Supplementary information.

## Data Availability

The data that support the findings of this study are available on request from the corresponding author. The data are not publicly available due to privacy or ethical restrictions.
