# Supplementary material for: GABA and glutamate measurements in temporal cortex of autistic children
Source: Autism Res. 2024 Nov 11;17(12):2558–71. doi: 10.1002/aur.3253 (PMC11638920; doi:10.1002/aur.3253)
Supplement: Supplementary file 1 — Data S1: Supplementary information. [file AUR-17-2558-s001.docx]

**Table S1:** Qualitative parameters

|  | **Left Hemisphere** | | **Right Hemisphere** | |
| --- | --- | --- | --- | --- |
|  | **ASD (N=46)** | **TD (N=29)** | **ASD (N=43)** | **TD (N=29)** |
| **CRLB (%)** |  |  |  |  |
| GABA | 24.3±40.5 | 15±6.4 | 9.6±2.2 | 10.5±3.6 |
| Glu | 7.7±1.8 | 7.2±1.1 | 8.8±2.2 | 7.6±1.0 |
| **SNR** | 23.6±4.8 | 24.6±3.9 | 23.5±4.9 | 23.9±3.1 |
| **Main magnetic field (B_0_) drift (Hz)** | –0.35±1.1 | –0.9±1.2 | –0.3±1.4 | –1.0±1.2 |
| **Linewidth at FWHM (Hz)** | 5.8±1.5 | 5.3±1.1 | 5.6±1.6 | 4.8±1.6 |
| **Metabolite concentrations (i.u.)** |  |  |  |  |
| GABA | 1.66±0.54 | 1.78±0.38 | 2.36±0.41* | 2.15±0.37 |
| Glu | 22.69±3.74 | 22.81±2.75 | 19.61±2.65* | 21.29±2.53 |

All values are expressed as mean±SD. Signal-to-noise ratio (SNR) and full-width half maximum (FWHM) were measured from the sum spectra; *p≤0.05 between the two groups within the region using ANCOVA


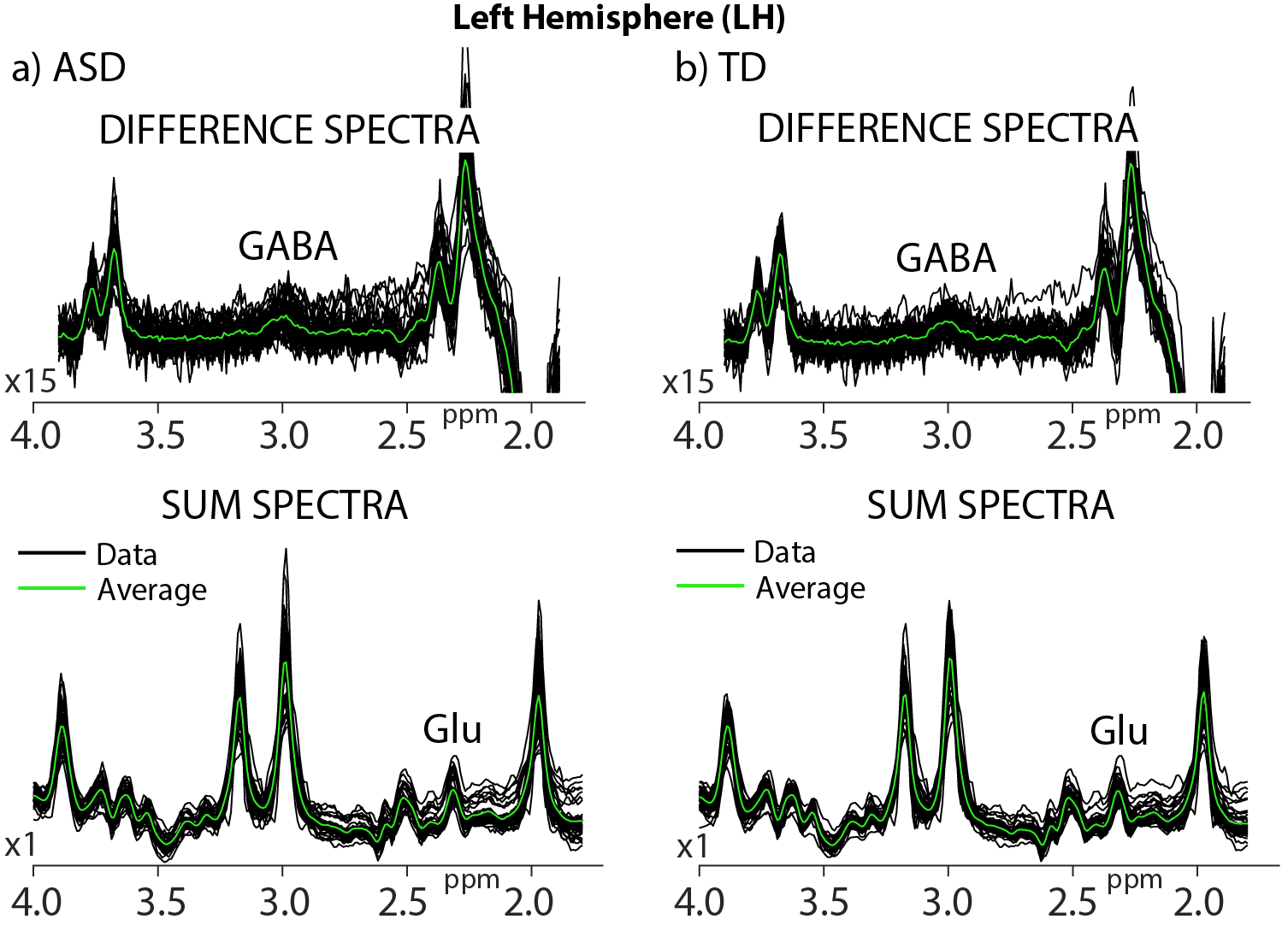


**Figure S1:** Individual difference (top) and sum (bottom) spectra acquired in the left hemisphere of the a) ASD and b)TD auditory cortices. The average spectra per group are in green. The difference spectra were scaled by a factor of 15 for visualization purposes only.


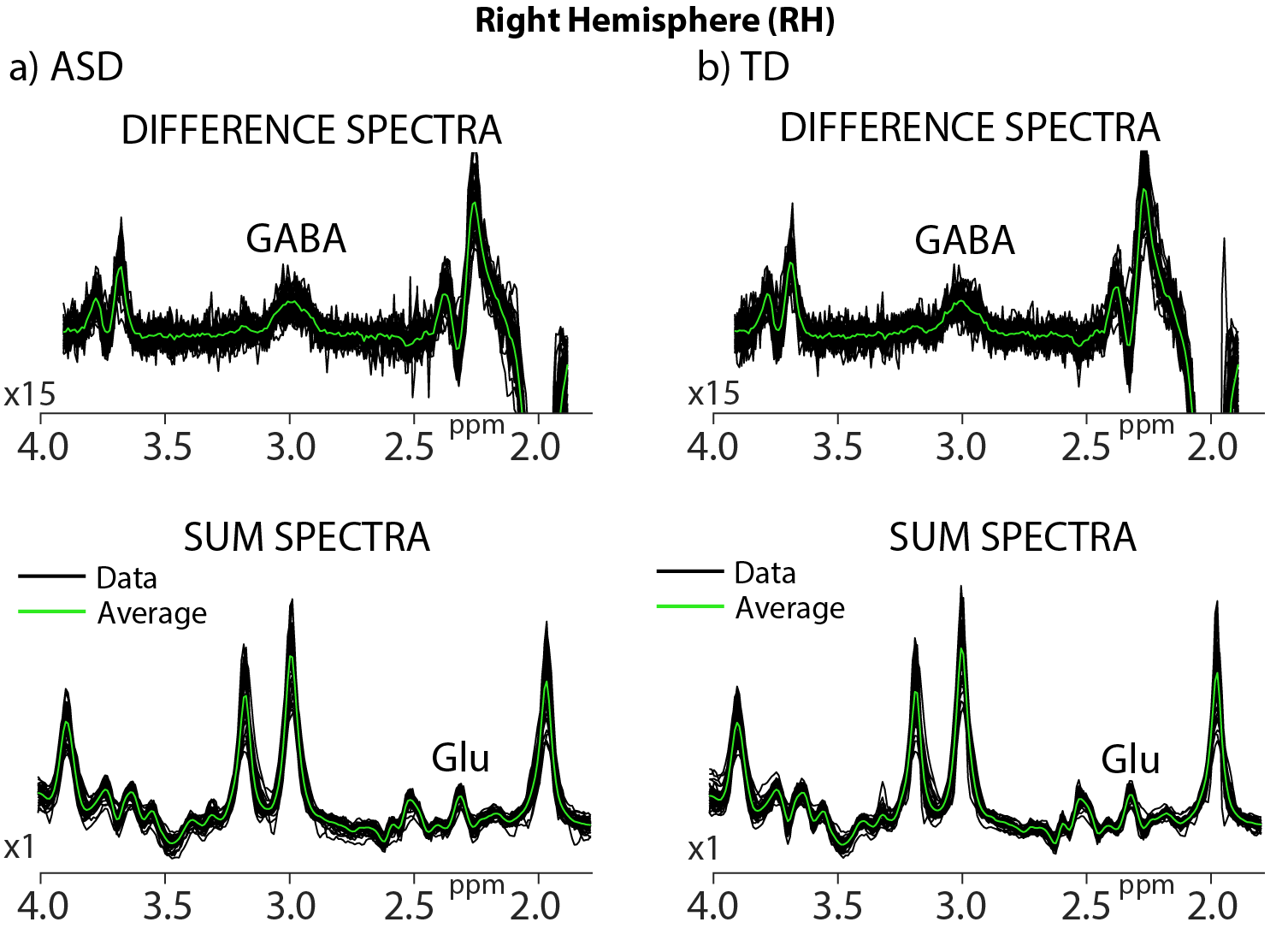


**Figure S2:** Individual difference (top) and sum (bottom) spectra acquired in the right hemisphere of the a) ASD and b)TD auditory cortices. The average spectra per group are in green. The difference spectra were scaled by a factor of 15 for visualization purposes only.


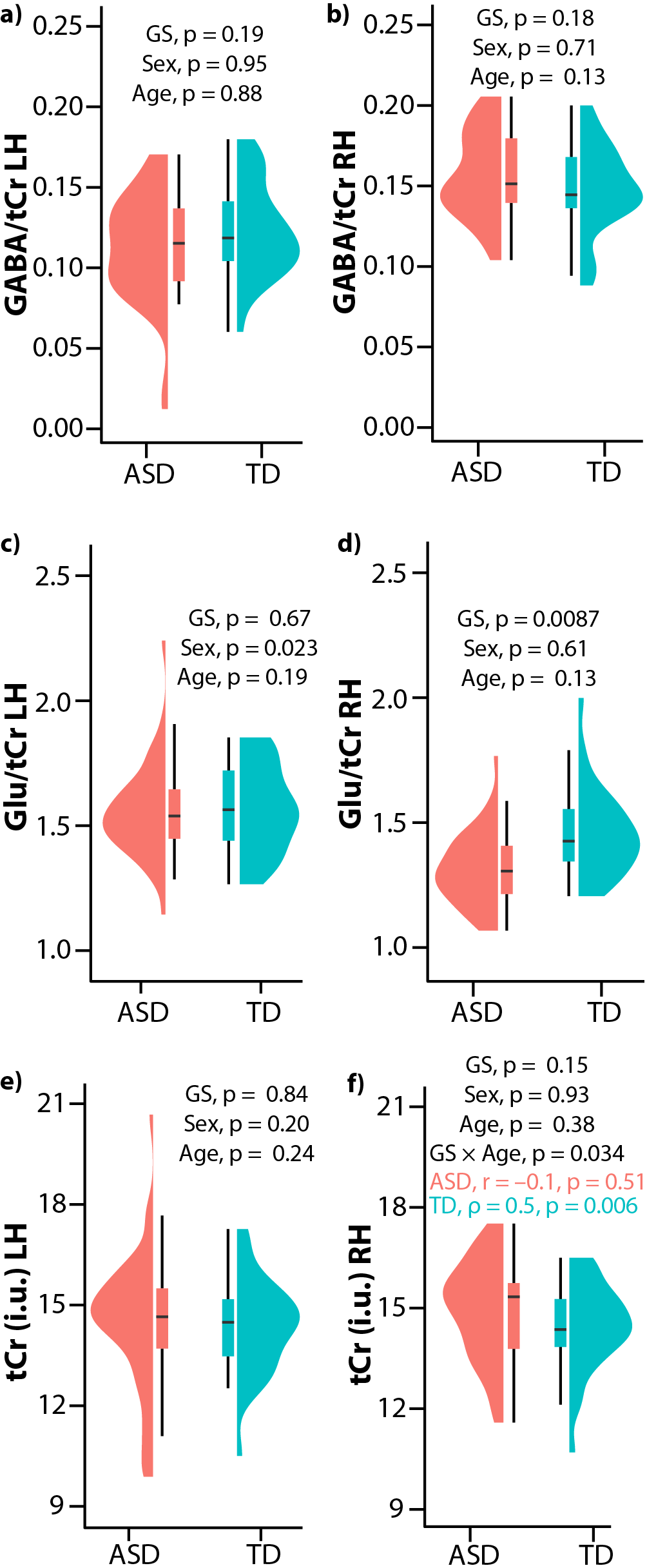


**Figure S3:** Raincloud plots of GABA/tCr (a and b), Glu/tCr (c and d), and tCr (e and f) in the left (left) and right (right) hemispheres. The statistical results are from running ANCOVA for every metabolite (separately) in each region with age and sex as covariates. Only (d) Glu/tCr in the right hemisphere is significantly different between the groups (p<0.05), whereas tCr positively correlates with developmental age (p<0.05). GS: Group Status; LH: Left Hemisphere; RH: Right Hemisphere; TD: Typically Developing; ASD: Autism Spectrum Disorder; i.u.: institutional units.; p-values for the GS, sex and age effects are from ANCOVA.


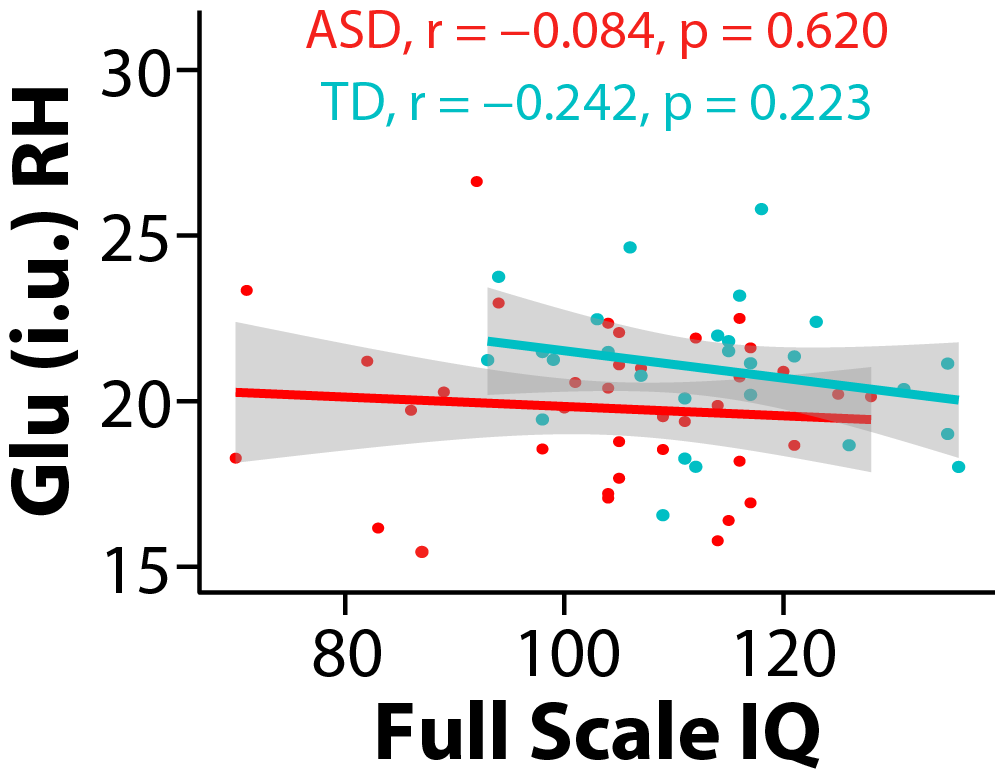


**Figure S4:** Glu correlations with Full Scale IQ (FSIQ) in the ASD and TD groups separately. RH: Right Hemisphere; TD: Typically Developing; ASD: Autism Spectrum Disorder; i.u.: institutional units.
